# Perceived social support in the daily life of people with Parkinson’s disease: a distinct role and potential classifier

**DOI:** 10.1038/s41598-025-12787-w

**Published:** 2025-07-24

**Authors:** Júlia Schönfeldová, Chen Cohen, Ortal Otmazgin, William Saban

**Affiliations:** 1 Center for Accessible Neuropsychology, Sagol School of Neuroscience, Tel Aviv University, Tel Aviv, 69978 Israel; 2 Department of Occupational Therapy, Gray Faculty of Medical & Health Sciences, Tel Aviv University, Tel Aviv, 69978 Israel

**Keywords:** Perceived social support, Parkinson’s disease, Cognition, Depression, Anxiety, Occupational health, Preclinical research, Translational research, Human behaviour

## Abstract

Motor outcomes in Parkinson’s disease (PD) have long been the primary diagnostic criteria and treatment targets. While non-motor outcomes of PD impact daily well-being, they are rarely targeted by interventions or utilized for classification. Despite promising evidence, the contributions of perceived social support (PSS) to PD detection and well-being in real-world settings remain unclear. Using remote monitoring technologies, we investigated the relationship between PSS and three non-motor measures—cognition, anxiety, and depression—in 92 participants: 45 PD and 47 matched-controls. To examine the specificity of PSS to non-motor features, we also examined the associations between PSS and three motor-related measures: disease severity, duration, or stage. Moreover, we developed machine-learning classifiers (ML) based on only non-motor features to identify disease status (PD/controls) in two cohorts: low and high PSS. PSS was significantly associated with non-motor measures in PD, with stronger correlations than in matched-controls in real-world settings. However, no significant correlations were found between PSS and the three motor-related measures, demonstrating PSS’s limitations. While the ML classification models performed low in high-PSS, they classified 13% better in a low-PSS cohort (AUC = 0.8), demonstrating moderate-high discriminatory performance. Taken together, our findings underscore the role of PSS in PD, highlighting its distinct contributions to non-motor classification models and the daily well-being of patients.

## Introduction

Despite its limitations, pharmacological interventions remain central to Parkinson’s Disease (PD) motor symptoms management^1–3^. Yet, addressing non-motor symptoms in people with PD (PwP) is vital for improving patient well-being in their real-world settings. The importance of social support for humans has been well-studied before^4,5^, including its promising positive impact on the well-being of PwP^6^. However, the potential contributions of Perceived Social Support (PSS) in the daily lives of PwP are poorly understood.

The COVID-19 pandemic highlighted the importance of social support in the day-to-day challenges of various populations, including PwP^7–11^. Nearly half of the PwP self-reported a negative change in PD symptoms during the COVID-19 pandemic^11^. When participants were asked to explain what factors might have contributed to the negative mood symptoms, the lack of physical contact with others emerged as the most quoted by PwP^11^. Given the impact of the COVID-19 pandemic on social interactions and the recent global adverse events, particularly in the Middle East, ongoing research into the role of social support is critical.

Social support can be a buffer against the mental and physical health consequences of illness or adverse life events^12^. In coping with PD-related challenges, the degree of social support appears fundamental. Indeed, social resources aid PwP in their day-to-day challenges, and they might even serve as resilience factors in symptom management^13,14^. Notably, PwP experience a complex combination of mobility challenges^15^, cognitive alterations^16^, and communication difficulties^17^, all of which could introduce social barriers and significantly affect the patient’s ability to engage in social interactions.

First, motor impairment in PD is associated with social withdrawal and can even predict it^18^. It was found that social withdrawal stems from both the mobility challenges and the fear of anticipating them^19^. The fear of falling is a common concern among PwP and it leads to avoidance behavior in social contexts^20^. Second, PD is associated with cognitive impairments that disrupt daily functioning^16^, and can interfere with social interactions. Third, social barriers may arise from communication challenges, which are derived from language and motor speech impairments. These symptoms hinder engaging in daily conversations, and PwP must often rely on cooperation with their communication partners^21^.

For example, hypomimia or dysarthria were identified as communication challenges that influence the social well-being of PwP^17,22^. Less expressive PwP may be deemed as cold or untrustworthy^23^. In addition, PwP have difficulty with recognizing the emotional expressions of others. It was found that PwP are less accurate in decoding positive and neutral facial expressions compared to controls^24^. Dysarthria is another prominent social symptom of PD with up to 89% of patients experiencing motor speech impairment^17,21^. This symptom was found to be a precursor of social withdrawal^18^.

Interestingly, while social support is known to be important, the perception of social support may also have a significant impact on an individual’s well-being^25,26^. PSS refers to the person’s subjective beliefs about the availability of social resources if needed^27^. Previous studies in PwP focused on social support, not taking into account PSS^6,28^, especially not in the patient’s daily life. PSS fundamentally differs from received social support in that PSS emphasizes the subjective perception of the support.

This distinction can be crucial. Studies demonstrated that PSS can be associated with more beneficial health outcomes compared to received social support^29,30^. For instance, in a large-scale (*n* = 1288) study, it was found that PSS had a weak association with received support^29^. In addition, greater PSS had a significant association with lower depressive outcomes^29^. However, greater received support had only a small association with lower depression, which was fully mediated by PSS^29^.

Moreover, the role of social support has been studied primarily by examining the associations within PwP without direct comparison to a control group^6,11,28,31^, limiting the ability to identify the unique contributions of social support to PwP. A control group can provide a comparison baseline to PwP with healthy individuals. Furthermore, previous studies have often considered only a limited set of factors. For example, studies examined only one or two outcomes, such as depression or anxiety, not considering the broader spectrum of both motor and non-motor outcomes in PwP^6,7,28,31^. This narrow approach restricts the development of a comprehensive understanding of how PSS contributes to various motor and non-motor outcomes of PD. A more comprehensive approach that examines both motor and non-motor outcomes, and compares PwP directly to a matched-control group, would provide a complete picture of the distinct role of PSS in PwP.

In addition, previous studies investigated social support in PwP mainly in clinical settings^14,28^. By utilizing remote online technologies, our study aimed to allow participants to report their PSS from the environment in which they regularly experience it. This approach is especially suitable for PwP, whose participation may be hindered in clinical settings due to mobility challenges^32–34^.

To conclude, the global context shaped by the post-COVID-19 pandemic highlighted the importance of PSS. In addition, although PSS might be associated with more beneficial health outcomes compared to received social support^29,30^, this distinction was not always made in previous PD studies^6,28^. Considering the above-mentioned unique challenges faced by PwP, PSS may play a crucial role in supporting the person’s daily well-being. We aimed to provide a comprehensive understanding of the selective role of PSS in motor and non-motor outcomes in PwP.

First, using online platforms, we examined the relationship between PSS and non-motor outcomes in individuals with PD and neurotypical healthy (NH) controls matched for age and education. We hypothesized that there would be significant associations between PSS and three non-motor outcomes: cognitive status, anxiety, and depression among the PD group. In line with previous studies^35^, with increasing PSS, we expected higher scores on the cognitive test, and lower scores on the anxiety and depression tests among the PD group. Importantly, given the unique combination of mobility challenges, cognitive impairments, and communication difficulties faced by PwP^16,17,36^—which suggests a selective dependency on PSS—we hypothesized that the associations between PSS and the non-motor outcomes would be stronger in the PD compared to the NH group.

In addition, in line with previous studies that found no significant associations between social support and motor-related outcomes in PD^37,38^, we hypothesized that there would be no significant correlations between PSS and motor-related outcomes in the PD group.

## Results

### Participants

The total sample size was 92 participants: 45 PwP and 47 NH controls. Table 1 shows the demographics, non-motor, and motor measures of both groups. There was no significant difference between the groups in age (t(85) = − 1.373, *p* = .173) and years of education (t(89) = -1.029, *p* = .306).

**Table 1:** Demographics, non-motor, and motor measures of both groups. Mean [SE].

Group	Age	Years of education	MoCA	ISEL-12	PROMIS-anxiety	PROMIS-depression
Neurotypical healthy	60 [1.9]	15.1 [0.3]	26.6 [0.3]	28.8 [0.8]	51.2 [1.1]	51.3 [1.1]
Parkinson’s disease	63.2 [1.4]	15.6 [0.4]	25.3 [0.4]	26.9 [1]	53.5 [1]	53.7 [1.2]

### Associations between PSS and non-motor outcomes in PwP and NH

First, we examined the differences between the groups in each non-motor measure (see Table 1). In terms of cognitive status (i.e., MoCA), the NH group received significantly higher scores compared to the PD group (t(81) = 2.478, *p* = .015). However, no significant differences were observed between the groups in all other non-motor measures (PSS: t(85) = 1.596, *p* = .114; PROMIS-anxiety: t(89) = -1.578, *p* = .118; or PROMIS-depression: t(88) = -1.528, *p* = .129).

**Fig. 1:**
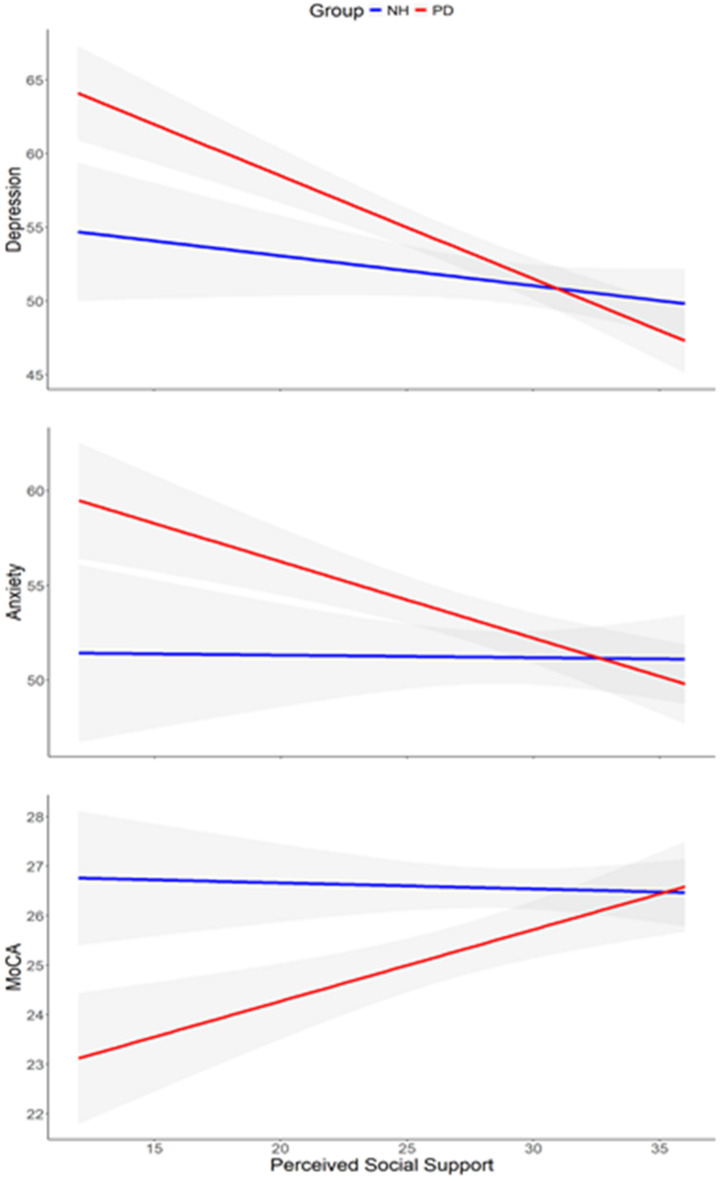
Associations between perceived social support (ISEL-12) and cognitive status (MoCA), anxiety (PROMIS-anxiety), and depression (PROMIS-depression) in the Parkinson’s disease (PD) and neurotypical healthy (NH) groups.

**Fig. 2:**
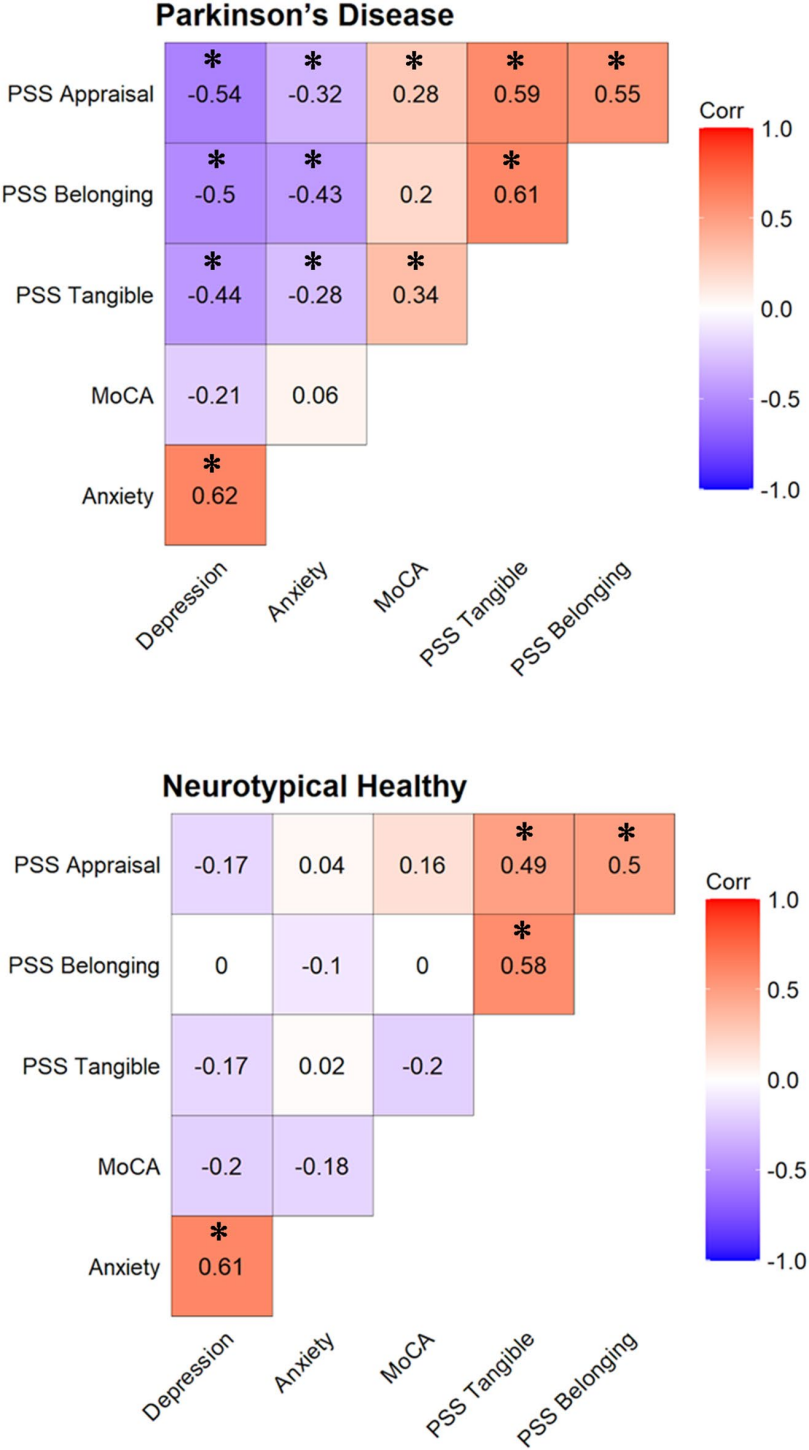
Associations between perceived social support (PSS) subscales and cognitive status (MoCA), anxiety (PROMIS-anxiety), and depression (PROMIS-depression) in both groups. *p < .05.

Second, we examined the associations between PSS and non-motor measures in each group (see Fig. 1 below). In the PD group, significant associations were found between PSS and the three non-motor measures (MoCA: *r* = .330, *p* = .013; PROMIS-anxiety: *r* = − .389, *p* = .004; PROMIS-depression: *r* = − .574, *p* < .0001). As expected, when PSS increased, the cognitive status increased, while anxiety and depression decreased. In addition, we found that these associations in the PD group were partially dependent on the PSS subscales: appraisal, tangible, and belonging (see Fig. 2 below). The cognitive status was significantly associated with tangible and appraisal PSS only. Depression and anxiety were both significantly associated with all three PSS subscales.

However, the associations between PSS and the three non-motor measures were not significant in the NH group (MoCA: *r* = − .030, *p* = .837; PROMIS-anxiety: *r* = − .009, *p* = .948; PROMIS-depression: *r* = − .145, *p* = .327). Accordingly, when examining the PSS subscales, all associations were not significant in the NH group (see Fig. 2 below).

Third, to examine the selective role of PSS in the PD group, we investigated whether the associations were significantly different compared to the NH group. Importantly, compared to the NH group, the PD group demonstrated significantly stronger associations between PSS and all measures (MoCA: z = 1.733, *p* = .041; PROMIS-anxiety: z = 1.86, *p* = .031; PROMIS-depression: z = 2.35, *p* = .009), demonstrating its selective association with non-motor outcomes of PwP.

### Associations between PSS and motor-related measures in PwP

**Fig. 3:**
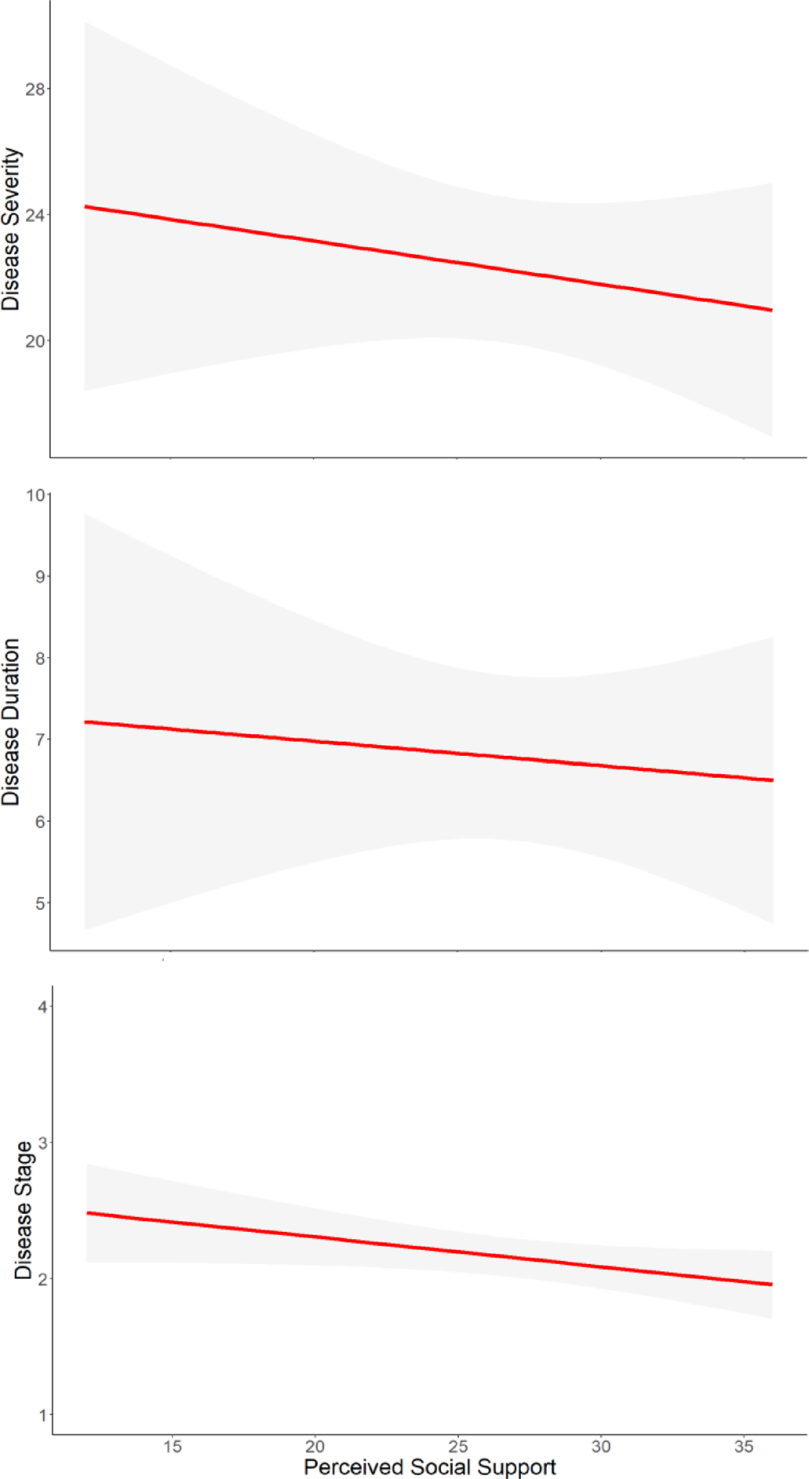
Associations between perceived social support (ISEL-12) and disease severity (MDS-UPDRS III), disease duration, and disease stage (H&Y) in the Parkinson’s disease group.

We then assessed the selective importance of PSS to non-motor outcomes, by examining the associations between PSS and motor-related measures within the PD group. See Figure 3 for the association between PSS and motor-related measures. No significant associations were found between PSS and motor-related measures in the PD group (MDS-UPDRS III: *r* = -.075, *p* = .625; disease duration: *r* = -.037, *p* = .808; H&Y: *r* = -.192, *p* = .207).

### Leveraging ML classification models and remote testing to identify PD using non-motor features

We then examined whether collecting non-motor data through online platforms could potentially provide valuable information for PD classification models. A comprehensive review of studies that applied computational techniques for the diagnosis of PD found 168 previous studies that distinguished PwP from NH controls^39^. Importantly, only 2 out of the 168 studies reported model performance results using non-motor measures alone^39^. Typically, studies that include non-motor measures in their classification models do so along with other motor, neuroimaging, and/or biofluid biomarkers^40–43^. Thus, studies that distinguish PwP from matched-controls based on non-motor measures alone are scarce.

If successful, remotely collected non-motor data—both accessible and scalable—could help improve PD screening tools. Notably, based on the correlational analysis, we investigated whether the performance of non-motor classification models could be dependent on varying levels of PSS. Given the observed PSS selective effects in PD, we hypothesized that classification model performance would improve at lower levels of PSS. We expected that in a cohort with high PSS, a classification model would exhibit low to moderate performance in distinguishing between PD and NH individuals. In contrast, among a low PSS cohort, we expected the classification model to demonstrate better performance, achieving moderate to high classification abilities. If supported, this pattern of results would suggest that the classification utility of non-motor features is modulated by PSS, indicating that PSS could serve as a potential classifier for PD.

**Fig. 4:**
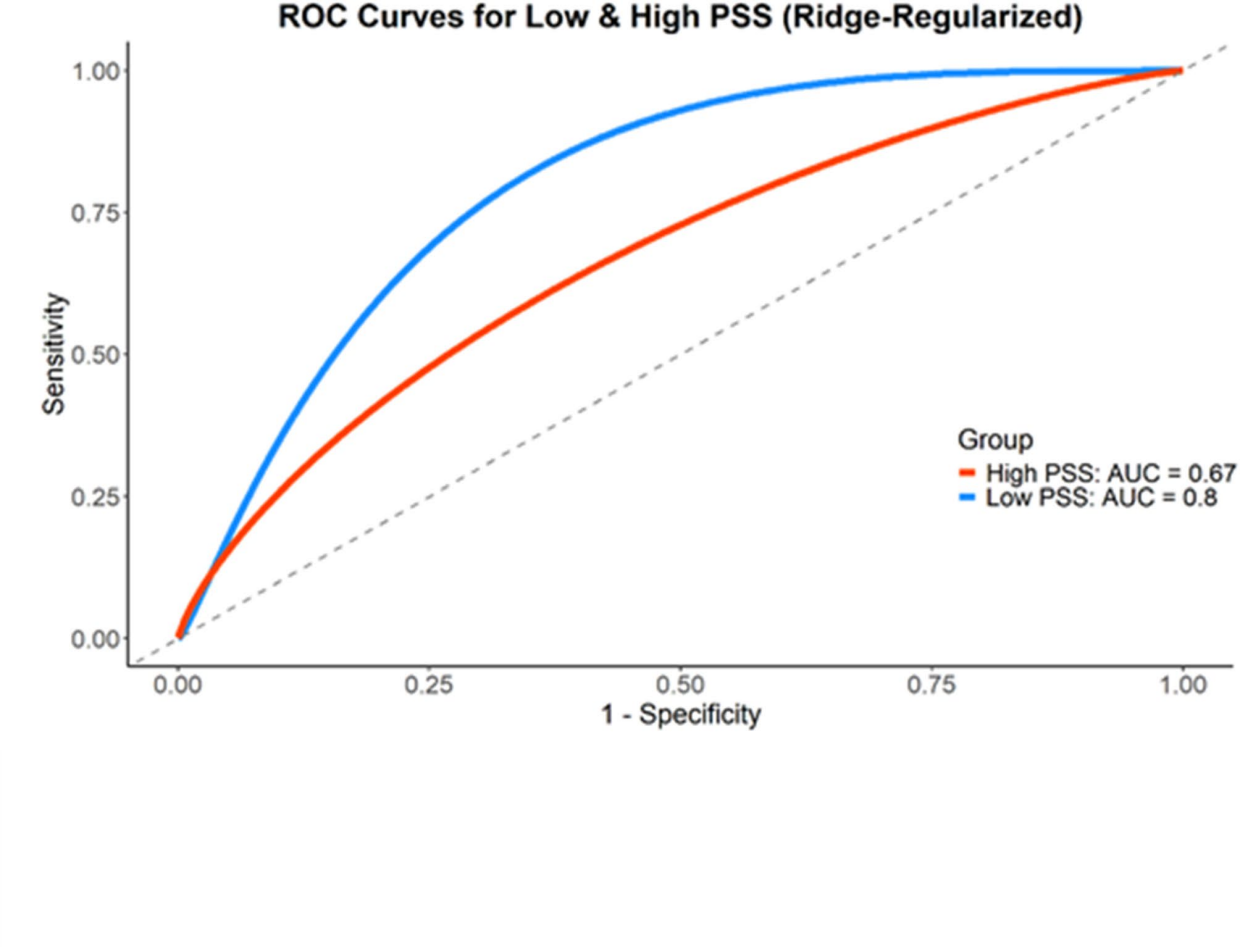
Receiver Operating Characteristic (ROC) curves for the classification of Parkinson’s disease vs. healthy matched control participants in high and low perceived social support (PSS). The ROC curve analysis revealed improved model performance at the low PSS cohort, with a higher (13%) Area Under the Curve (AUC) compared to the high PSS cohort.

Accordingly, we divided the dataset into two cohorts based on the median of the ISEL-12 total scores, categorizing participants into high and low PSS cohorts. We developed ML classification models using non-motor features to differentiate between individuals with PD and NH controls (see Methods). The results of the classification models are presented in Fig. 4. For the high PSS cohort, the ML classification model achieved an Area Under the Curve (AUC) of 0.67 and an accuracy of 0.49 (precision = 0.29, recall = 0.1, F1-score = 0.15), reflecting low discriminatory power, and the ML bias toward predicting the NH group. However, in the low PSS cohort, the classification model achieved moderate-high discriminatory power, with an AUC of 0.8 and an accuracy of 0.72 (precision = 0.77, recall = 0.68, F1-score = 0.72), effectively distinguishing PD from NH individuals based on non-motor features.

To statistically compare the MLs’ classification performance between the two PSS cohorts, we calculated the difference in observed AUCs between the low and high-PSS cohorts. To assess the significance of this difference (13%), we computed an empirical distribution of AUC differences by subtracting AUCs from 1,000 permutation-based null models in each cohort. The observed difference exceeded all values in the null distribution, yielding an empirical *p* < .0001, indicating a statistically significant and robust performance gap between the two PSS cohorts. This result provides strong evidence that the ability to distinguish PD from NT participants using non-motor features was significantly greater under conditions of low-PSS than under high-PSS.

Also, when examining accuracy, the results reflect a 23% improvement in classification accuracy (72% vs. 49%) in the low-PSS cohort relative to the high-PSS cohort, highlighting the potential role of PSS in the detectability of non-motor differences between PD and NT individuals. Taken together, these findings suggest that ML classification models perform better in individuals with low levels of PSS compared to those with high levels. In addition, the classification models are effective at distinguishing between PD and NH individuals using non-motor features alone.

## Discussion

We hypothesized that the associations between perceived social support (PSS) and non-motor outcomes would be more pronounced in people with Parkinson’s disease (PwP) compared to neurotypical healthy (NH) controls. This hypothesis was predicated based on the unique combination of day-to-day challenges faced by PwP^15–18^, including mobility limitations, cognitive alterations, and communication difficulties, which can explain the specific role of PSS in this clinical population.

In support of this hypothesis, we found stronger associations between PSS and cognitive status, anxiety, or depression in the PD group compared to the NH group. Accordingly, we observed that while most of the associations between the PSS subscales and all the non-motor outcomes were significant in PwP, none were significant in the NH group. Additionally, no associations were found between PSS and motor severity, disease duration, or disease stage in the PD group. This pattern of results highlights the selective importance of PSS for non-motor outcomes of PD. This nuanced approach aims to delineate the specific contributions – and limitations – of PSS for PwP.

The negative relationship between PSS and both depression and anxiety in PwP was also found previously^6,28,31^. However, one concern is the lack of association between PSS and the non-motor measures in the NH group. This lack of significant correlations may be due to the limited sensitivity of the tools (i.e., ISEL-12, PROMIS, MoCA) used in the current study. These measures may not be sufficient to detect subtle associations within a healthy population, as they are short (< 10 min) and rough estimates for non-motor domains. Future research could employ more comprehensive and sensitive instruments to assess these non-motor measures and PSS. However, one should notice that longer tools may be less feasible for accessible, online administration.

Interestingly, the association between PSS and motor measures in PwP was previously investigated in a limited manner. One study investigated the association between social isolation and PD symptom severity in general^10^. The study utilized the PROMIS-PD questionnaire, which measures 33 PD symptoms across motor, autonomic, cognitive, and emotional domains, all through the participants’ self-reports^10^. It was found that more social isolation was correlated with worsened PROMIS-PD scores. In addition, lonely PwP reported greater symptom severity on all 33 PROMIS-PD items than non-lonely PwP^10^. Of note, this study measures the participant self-reports, so it is unclear whether social support can directly affect the motor symptoms of PwP. Another study measured the motor-related outcomes in PD through the objective measure of UPDRS-III and reported no significant association with social support^38^. UPDRS-III score was also found to be positively correlated with the total score of the PDQ-39, which includes a social support subscale^37^. Interestingly, both the UPDRS-II and UPDRS-III measures were not correlated with social support^37^. Accordingly, our current results do not support the direct or indirect relationship between PSS and PD motor outcomes.

Nevertheless, there is a shared biological pathway simultaneously influencing social perception and motor control. For instance, the limbic system, encompassing structures such as the amygdala and hippocampus, is integral to both emotional processing and motor functions^44^. This suggests that social support could potentially modulate emotional responses and also promote motor function by engaging this interconnected network. Additionally, the dopamine system, which is involved in reward processing and social interaction regulation^45^, may theoretically link social support to motor control. While our findings do not support this association between PSS and motor-related measures, the potential of PSS to influence motor function through these suggested mechanisms is theoretically possible. The lack of a significant correlation in our study and others may be attributed to limitations such as measurement sensitivity, rather than a true null effect.

PD served us as a useful test case primarily due to its high prevalence as the most common motor neurodegenerative disorder. Given that we compared the PD group only to the matched neurotypical group, we could not determine whether the observed associations between PSS and non-motor outcomes are specific to PD or generalize to other neurological conditions. Future research should include relevant clinical groups to clarify whether the impact of PSS is unique to PD or reflects a broader pattern across neurological disorders.

Importantly, we leveraged ML classification models and remote testing platforms to identify whether a participant is PD or NH based on non-motor features only. The computational findings suggest that classification models perform better by 13% in individuals with low levels of PSS compared to those with high levels of PSS. In addition, this classification model is moderately to highly effective (AUC = 0.8) at distinguishing between PD and NH individuals using non-motor features alone. Thus, we found that the classification utility of non-motor features is modulated by PSS, suggesting that PSS could serve as a potential classifier for PD.

Our study demonstrates that collecting non-motor measures through online platforms provides valuable information for classification models. Using non-motor features alone, these computational models can significantly distinguish between PD and matched-controls remotely. Thus, remotely collected non-motor data could enhance tools for PD screening. Using large online databases, we propose that future studies examine the predictive power of PSS and other non-motor measures as potential predictors in an independent dataset.

Lastly, we suggest that PD-dedicated interventions will focus on ensuring that PwP have opportunities to discuss their challenges and confide in family, partners, or healthcare professionals. Importantly, we recommend that the interventions will focus on the subjective interpretation of social support.

## Conclusions

To summarize, our results demonstrated that PSS is uniquely linked to non-motor outcomes in PD. Additionally, remote and accessible PSS assessments can help identify PD subgroups with emotional and cognitive alterations. Integrating remote PSS evaluations with ML models could potentially offer an accessible and effective method for PD classification. We propose that incorporating an accessible PSS measure into digital-health platforms can be used as a non-motor-marker to identify at-risk PD subgroups and guide more targeted, modifiable interventions. The results underscore the need for targeted interventions aimed at enhancing the perception of social support as a promising method to improve non-motor outcomes of PwP.

## Methods

This was a prospective, observational, cross-sectional study with automated administration and scoring to minimize inter-rater variability both in terms of administration and scoring. For the remote evaluations, we followed the published protocol for online neuropsychological testing (called iPONT)^33,34,46–49^. Both the PD and NH groups were recruited through our clinical Center for Accessible Neuropsychology (CAN) database. The database is comprised of individuals who were tested in CAN before or who have responded to online advertisements in PD-dedicated associations, such as the “Israel Parkinson Association.” Inclusion criteria required a minimum of 18 years of age, fluency in Hebrew, normal or corrected vision, access to the computer and camera, and the ability to complete questionnaires online. Individuals with other neurological conditions (not PD), psychiatric conditions (besides anxiety and depression), learning disabilities, and severe visual or auditory impairments, were not included in the study. For the PD group, we did not include individuals with past surgical interventions (e.g., DBS), and all participants were tested while on their current medication regimen. All PwP had Hoehn and Yahr (H&Y) scores smaller than five. The PD group mean MDS-UPDRS-III was 22.2 ± 1.8 and their disease duration was 6.8 ± 0.8. This protocol was approved by Tel Aviv University Institutional Review Board committee, and all methods were performed in accordance with relevant guidelines and regulations.

Individuals were invited by email to participate in an online interview with the experimenter. Participants provided informed consent and completed a demographic and medical questionnaire. The questionnaires consisted of information related to the participant’s medical history, age at diagnosis, medication, primary symptoms, genetic subtype, diet, or other neurological or psychiatric conditions. Then we administered the Montreal Cognitive Assessment (MoCA)^46–50^ test to evaluate cognitive status. PwP were additionally assessed using the Movement Disorder Society – Unified Parkinson’s Disease Rating Scale (MDS-UPDRS III scale)^51^ and the H&Y scale^52^ as measures for disease severity and disease stage. Afterward, three self-reported questionnaires were sent via email to all participants who met the inclusion criteria: Patient-Reported Outcomes Measurement Information System (PROMIS) scales for anxiety and depression^53^, and the Interpersonal Support Evaluation List (ISEL-12)^54,55^. All questionnaires were administered via Google Forms.

The order of the questionnaires was randomized to avoid order effects. The session took 40–60 min to complete.

### Outcome measures

The MoCA^47–50^ test was used to evaluate cognitive status. MoCA has been widely used in clinical and research settings for cognitive screening in PwP^49,56^ and it was found to be more sensitive for assessing mild cognitive impairment compared with the Mini Mental State Examination^57^. PSS was measured using the ISEL-12 questionnaire with 12 items, which is a widely used measure for PSS^54,55,58^. Participants indicated the perceived availability of social support in their lives on a 4-point scale ranging from “definitely false” to “definitely true”. The ISEL-12 range of scores is 12–48, where higher reflects more support. The ISEL-12 measures PSS across three subscales: (1) Appraisal Support (perceived availability of someone to talk to, confide in, or seek advice from when facing challenges); (2) Tangible Support (perceived availability of material or practical assistance, such as help with tasks or resources in times of need); (3) Belonging Support (perceived availability of companionship, social integration, and a sense of community or group affiliation). Each subscale consists of 4 items.

Anxiety and depression were measured using the PROMIS scales for anxiety and depression (PROMIS-anxiety and PROMIS-depression)^53,33^. The items are rated on a scale from 1 (never) to 5 (always). Higher scores indicate greater anxiety or depression. The PROMIS-anxiety and PROMIS-depression scales assess the severity of anxiety and depression using T-scores, which have a population mean of 50.

In terms of the three motor-related measures, motor symptom severity (range: 0-132) was evaluated through the MDS-UPDRS III^51^. Disease duration was reported by the participant based on the initial diagnosis by a movement disorder specialist. The disease stage (range: 0–5) was measured by the H&Y scale^52^.

### Statistical analysis

First, we conducted a power analysis (alpha = 0.05; power = 0.99) to calculate the required sample size. Effect sizes were derived from five studies that compared PD and NH groups^59–63^ (D = -1.152). The analysis proposed a minimum of 14 participants per group, making the sample sizes of our groups (> 40) sufficient for detecting group differences.

For examining the score differences between the groups on PSS, cognition, anxiety, and depression scores, an independent-sample t-test was used. For associations between PSS scores and non-motor/motor outcomes in the PD or NH groups, Pearson’s r test was utilized. Fisher’s z-test was used to compare the strength of the associations between groups.

We also developed ML classification models using Generalized Linear Models (GLMs, logistic regression), to differentiate between individuals with PD and NH matched-controls. We applied L2-regularized logistic regression (Ridge) with cross-validation to classify participants as PD or NH within each PSS cohort (low and high), using the non-motor predictors. The regularization parameter (λ) was tuned within each training fold (low PSS: average λ across folds was 0.34, high PSS: average λ across folds was 0.55). To assess the ML model’s generalizability, we applied 10-fold cross-validation, a procedure in which the dataset is randomly divided into ten equal-sized folds. In each iteration, the ML model is trained on nine folds and tested on the remaining one, ensuring that every observation serves as a test case. The classification model predictor variables included cognition, anxiety, depression, and age. To ensure unbiased comparisons and standardized feature scaling, all predictors were normalized using Z-score transformation. Z-score normalization was applied within each training fold, preventing information leakage.

All statistical analyses were conducted in R software^64^.

## Data Availability

The data is available upon reasonable request from the corresponding author. Access will be granted in compliance with ethical guidelines, given the patients’ confidential information.
